# Feasibility of epicardial implantation of medtronic 3830 lead in a pediatric patient : case report

**DOI:** 10.1186/s13019-024-02836-2

**Published:** 2024-07-20

**Authors:** Dou Yuan, Ke Lin, Yuanning Xu

**Affiliations:** Department of Cardiovascular Surgery, Cheng Du Shang Jin Nan Fu Hospital, West China Hospital, Sichuan University, Sinchuan Province, 610041 Shandong China

**Keywords:** Noonan syndrome, Modified Konno procedure, Permanent epicardial pacemaker, Children. Medtronic 3830 pacing lead, Case-report

## Abstract

**Background:**

High-grade atrioventricular block is the primary reason for epicardial permanent pacemaker implantation during the perioperative period in patients with congenital heart disease. Due to the smaller diameter of venous vessels in children, epicardial permanent pacemaker implantation is usually a preferred choice, we report one pediatric patient who received epicardial permanent pacemaker implantation using a new approach.

**Case presentation:**

We present the case of a 2-year-old girl who underwent the modified Konno procedure and Pulmonary valvuloplasty surgery and presented after surgery with a High-grade atrioventricular block. At over 20 days after the patient underwent a redo-sternotomy which epicardial permanent pacemaker implantation. Medtronic Model 4965 Capsure Epi ® steroid-eluting unipolar epicardial pacing lead was immobilized on the surface of the right ear. The Medtronic 3830 pacing lead was screwed obliquely and clockwise under direct view from the surface of the right ventricle to the endocardium near the interventricular septum. The patient’s recovery was uneventful.

**Conclusion:**

In this case report, we demonstrate the feasibility and potential benefits of using the Medtronic 3830 lead for epicardial pacing in a pediatric patient with severe cardiac complications following surgery for congenital heart disease. This approach offers a viable alternative to traditional epicardial pacing methods, particularly in complex cases where conventional leads fail to provide stable pacing thresholds.

## Introduction

The incidence of high-grade atrioventricular block(AVB) following surgery for congenital heart diseases (CHDs) is approximately 1%; This condition represents one of the primary indications for the implantation of epicardial permanent pacemakers during the perioperative period. The management of high-grade AVB is critical, as it can severely impact the postoperative recovery and long-term cardiac function of patients with CHD.

[1, 2]. Due to the smaller diameter of venous vessels in children, epicardial permanent pacemaker implantation is usually a preferred choice [3, 4]. However, children with CHD often require multiple operations, resulting in a high threshold of conventional pacing electrodes through epicardial pacing [5]. In our case report, we discuss the implementation of the Medtronic 3830 lead for epicardial pacing in a pediatric patient who developed high-grade AVB post-CHD surgery. Our focus is on assessing the feasibility and safety of this approach, which, though traditionally used for endocardial pacing, has shown promise in the epicardial setting under challenging conditions marked by extensive surgical scarring and fibrotic cardiac tissue.

## Case presentation

**Fig. 1:**
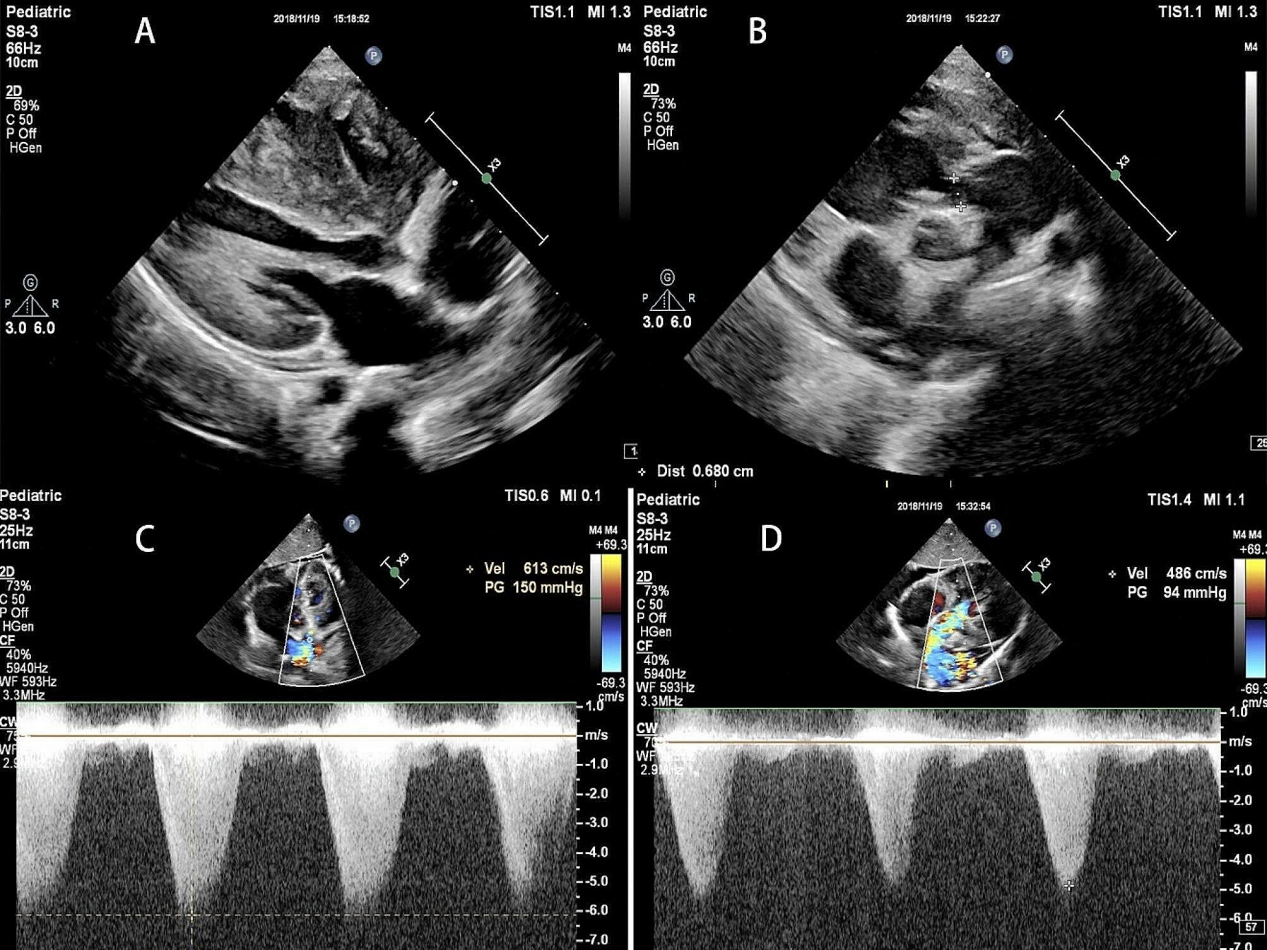
(**A**) Bilateral ventricular hypertrophy, RVAW: 9 mm, IVS: 13 mm, LVPW: 10 mm; supraventricular crest hypertrophy; (**B**) the inner diameter of the narrowest site under the pulmonary valve was about 4.6 mm; the circumferential diameter of the pulmonary valve was about 6.8 mm; (**C**) severe subvalvular and valvular pulmonary stenosis (Vmax = 6.1m/s, PG = 150mmHg); SAM phenomenon of anterior mitral valve leaflet in systole; (**D**) blood flow acceleration in the left ventricular outflow tract (Vmax = 4.8m/s, PG = 94mmHg); mild mitral regurgitation

**Fig. 2:**
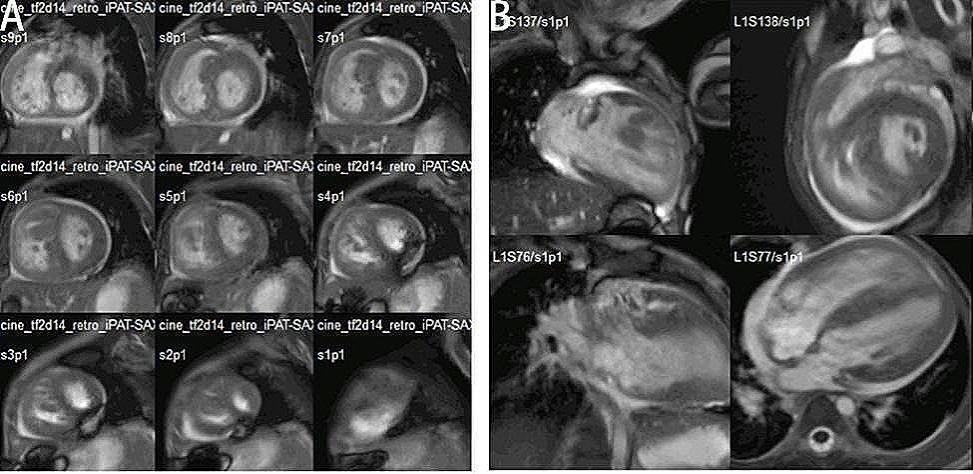
Cardiac MRI: ventricular hypertrophy

**Fig. 3:**
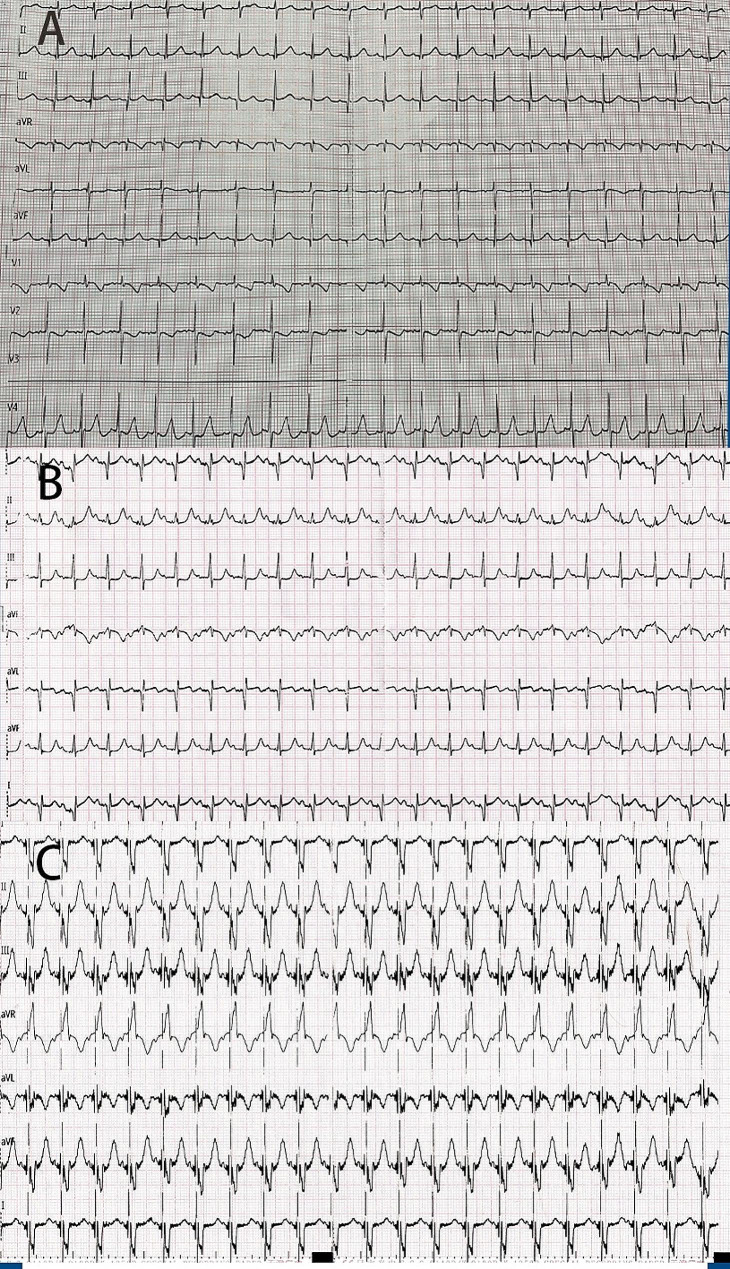
(**A**) Normal ECG (**B**) III AV Block, temporary pacemakers rely on ECG, (**C**) The dual-chamber pacemaker rely on ECG

A 2-year-old girl was admitted to the hospital due to murmurs found by physical examination 2 years earlier and perioral cyanosis after crying for over 1 month, the saturation is about 85%. The diagnosis of Noonan syndrome (NS), Hypertrophic obstructive cardiomyopathy (HOCM), Severe subvalvular and valvular pulmonary stenosis, Bilateral ventricular hypertrophy, and Mild mitral regurgitation were made by Transthoracic echocardiography (TTE, Fig. 1), the pressure gradient across the pulmonary valve was measured to be 150 mmHg, indicating severe pulmonary stenosis. This measurement was crucial for our surgical decision-making process, as it underscored the need for immediate intervention to alleviate the obstruction and restore normal hemodynamics. Cardiac Magnetic Resonance Imaging (MRI) (Fig. 2), Electrocardiography (ECG) (Fig. 3A), Genetic testing (PTPN11 gene mutation, Noonan syndrome type I, OMIM: 163,950).

**Fig. 4:**
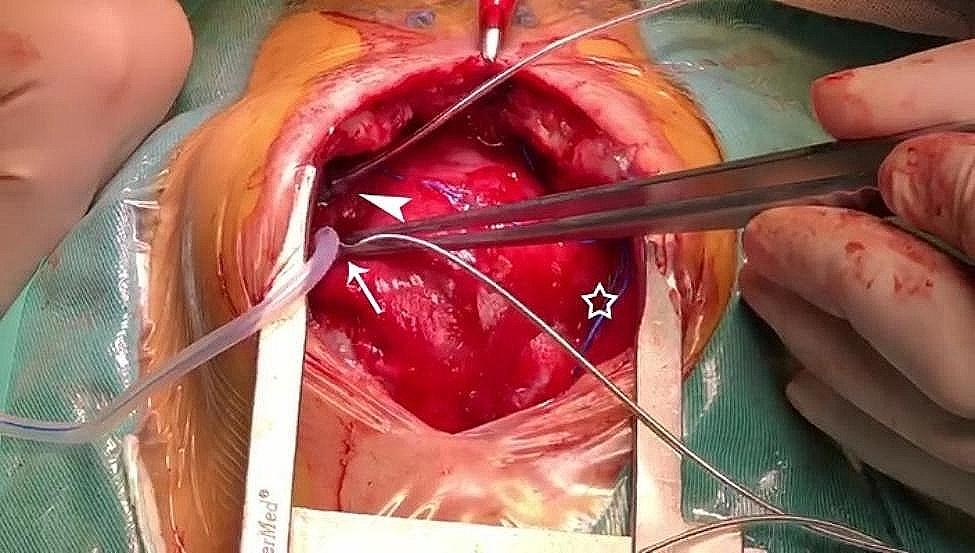
Medtronic Model 4965 Capsure Epi ® steroid-eluting unipolar epicardial pacing lead(arrowhead): The ventricular electrode was placed repeatedly at different positions on the ventricular surface, ( 4.0 V @ 0.4 ms, 2.6 V @ 0.4 ms, 6.5 V @ 0.4 ms).(**B**) TheMedtronic 3830 pacing lead (arrow)was screwed obliquely and clockwise under direct view from the surface of the right ventricle to the endocardium near the interventricular septum and immobilized (1.0 V@0.4ms).(**C**) Temporary pacing lead(pentagram)

**Fig. 5:**
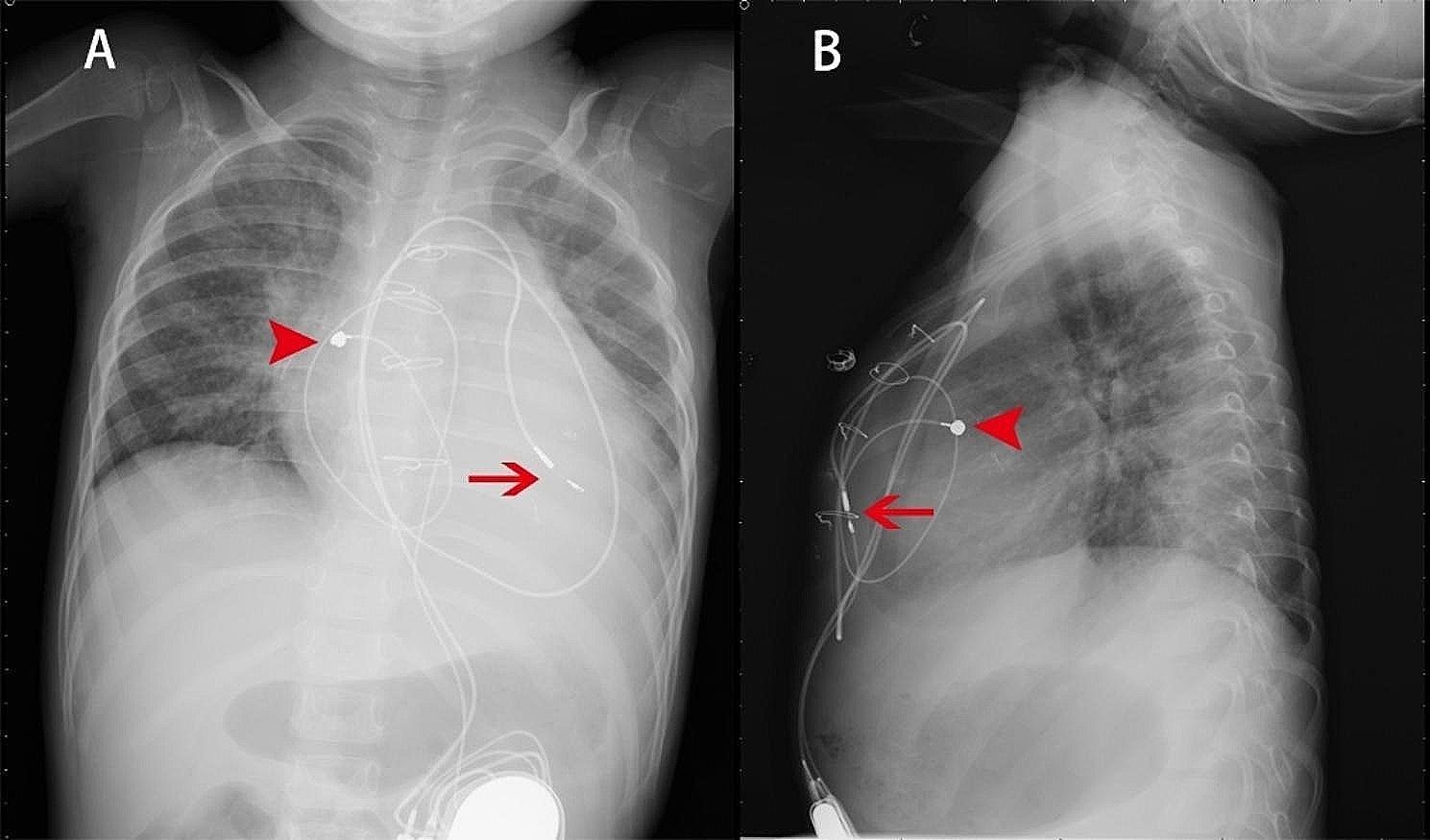
Medtronic Model 4965 Capsure Epi ® steroid-eluting unipolar epicardial pacing lead(arrow), The Medtronic 3830 pacing lead (arrowhead)

The operative team evaluated the patient before the surgical, afterward, a standard median sternotomy incision is performed. First surgery: The modified Konno procedure and Pulmonary valvuloplasty (PVP) and Temporary epicardial pacing lead placement; After surgery, the patient had a 3rd degree AVB (Fig. 3B). At over 20 days after the primary surgery: Epicardial permanent pacemaker implantation was performed under general anesthesia and tracheal intubation; During operation: The chest was opened along the original mid-sternal incision to expose the heart (Fig. 4). A pursestring suture was placed around the intended site of the lead penetration to ensure hemostasis and secure the lead placement. Medtronic Model 4965 Capsure Epi ® steroid-eluting unipolar epicardial pacing lead (4965Capsure Epi ®, Fig. 4, arrowhead) was immobilized on the surface of the right ear. Testing: pacing threshold 1.0 V, P-wave amplitude 1.3 mV, impedance 692 Ω. During the procedure, we encountered specific challenges that required immediate and careful management (4965 Capsure Epi ®). The ventricular electrode was placed repeatedly in different positions on the ventricular surface, and the pacing parameters were not satisfactory during testing (threshold 4.0 V @ 0.4ms, 2.6 V @0.4ms, 6.5 V @ 0.4 ms). At last, the Medtronic 3830 pacing lead (Fig. 4, arrow) was screwed obliquely and clockwise under direct view from the surface of the right ventricle to the endocardium near the interventricular septum and then immobilized (threshold 1.0 V @ 0.4ms ). When the testing results were satisfactory, the pacemaker generator was positioned in the posterior rectus sheath. This lead was connected to the Medtronic dual-chamber pacemaker. ECG (Fig. 3C), chest X-ray scan (Fig. 5) and pacemaker programming were performed for reexamination.

## Dicussions

NS is a common autosomal dominant disease, with up to 80% of affected individuals developing CHD, primarily PS, atrial septal defects, and HCM [6, 7]. For the pediatric patient reported in this study, a modified Konno procedure, PVP surgery was performed to completely relieve the outflow tract obstruction. However, this patient had a grade III AVB after surgery and was indicated for epicardial permanent pacemaker implantation.

The implantation of pacemakers in children can be divided into endocardial implantation and epicardial implantation [5]. While both epicardial and endocardial pacing are viable options for pediatric patients, each method has its distinct advantages and challenges, particularly in the context of congenital heart disease and post-surgical conditions. In this case, the epicardial approach was selected due to anatomical considerations and the patient’s specific clinical scenario, offering a practical solution where traditional methods were less feasible. The disadvantage of epicardial implantation is that their pacing threshold is unstable, and their pacing lead is easily oppressed by bone and muscle, resulting in wear and even fracture. In our case, the infant presented with unique anatomical and post-operative challenges that necessitated the choice of a screw-in lead designed for endocardial use. Due to the infant’s small size and the extensive cardiac remodeling following surgery for congenital heart defects, traditional epicardial leads were not providing stable or sufficient electrical parameters. The severe scarring and presence of fibrous tissue over the epicardial surface further complicated the situation, making it difficult to achieve and maintain effective pacing thresholds with typical epicardial leads.

Moreover, the decision to place the lead near the interventricular septum rather than the apex was driven by the goal of optimizing cardiac function while maintaining a lower pacing threshold. This approach was chosen despite it not being the typical first choice in infants due to the atypical cardiac anatomy and the specific challenges posed by the patient’s condition post-surgery.

In this patient, to perform implantation, the chest was opened along the original mid-sternal incision to better expose the atria and ventricles while achieving effective suturing and immobilization of the epicardial pacemaker electrode. As planned, one Medtronic Model 4965 Capsure Epi® steroid-eluting unipolar epicardial pacing lead was placed on the surface of the right atrium and the left ventricle under non-extracorporeal circulation, followed by the placement of a dual-chamber pacemaker. Undoubtedly, the dual-chamber pacemaker can maintain normal sequential atrial and ventricular pacing that mimics a physiological state [8]. However, some contingencies occurred during placement of the ventricular electrode. Although the components on the cardiac surface were already sufficiently dissociated and the ventricular electrode was placed repeatedly at different positions on the ventricular surface, the pacing parameters were not satisfactory during testing (threshold 4.0 V @ 0.4 ms, 2.6 V @ 0.4 ms, 6.5 V @ 0.4 ms). This may have been related to the fact that epicardial permanent pacemaker implantation was not performed until long after the surgery. For iatrogenic injury after surgery, it is generally believed that the pacemaker should be implanted after 7–14 days [9]. This may have been related to the fact that epicardial permanent pacemaker implantation was not performed until long after the surgery, which is known to increase the risk of scar formation and higher pacing thresholds. Since many Chinese patients are unwilling to receive permanent pacemaker implantation, this interval is usually longer than it should be, which can lead to complications such as scar and fibrous tissue formation. These post-surgical changes contribute to a high capture threshold due to the presence of scars and fibrous and hyperplastic tissues after extensive inflammatory responses on the cardiac surface, compounded by the high impedance of the epicardium itself. Satisfactory pacing parameters (threshold 1.0 V @ 0.4 ms) were finally obtained after the placement and immobilization of the Medtronic 3830 pacing lead on the surface of the right ventricle near the interventricular septum. The selection of the ventricular pacing site is a critical aspect of our protocol. Our primary considerations are in the following order: minimizing the pacing threshold, ensuring optimal cardiac function, and then considering the effects on QRS duration. In our case, the lead placement near the interventricular septum was chosen to optimize pacing threshold and cardiac output, as traditional apical positioning was not viable due to anatomical and post-surgical constraints. The benefits of this procedure can be summarized as follows: (1) There are many sites available for its placement, and there is no requirement for a smooth cardiac surface to ensure the desired pacing threshold; (2) the electrode can be screwed in further away from the phrenic nerve and diaphragm, thus avoiding postoperative stimuli to the diaphragm; (3) a high pacing threshold, large output current and fast battery loss can be prevented, which would otherwise be caused by a loose immobilization of the conventional epicardial pacemaker electrode; (4) the pacemaker electrode is placed into the myocardium and closer to the endocardium, such that the pacing more resembles endocardial pacing. The potential drawbacks of using the Medtronic 3830 lead for epicardial pacing in pediatric patients, which includes: (1) The placement of a lead designed primarily for endocardial use into the epicardial space may lead to an increased risk of fibrous tissue development around the lead over time. This could potentially increase the pacing threshold and require future surgical interventions (2). The procedure requires precise surgical skill to place the lead correctly without damaging surrounding cardiac tissue. The oblique and clockwise screwing of the lead into the myocardium is a delicate process that can potentially lead to complications such as cardiac perforation or tamponade if not performed meticulously (3). While short-term results may show satisfactory pacing thresholds and clinical improvement, the long-term stability and reliability of this off-label use of the lead are not yet established. Further studies and follow-up are required to validate its efficacy and safety over time.

The use of the Medtronic 3830 lead, typically designed for endocardial use, for epicardial application in our case was unconventional. The potential risks including the possibility of increased fibrosis and rising capture thresholds over time. A long-term follow-up is necessary to validate the safety and efficacy of this method. So we will commit the follow-up plan for this patient and the need for ongoing monitoring to assess long-term outcomes and complications.

To the best of our knowledge, this report is the first to describe the use of the Medtronic 3830 lead for epicardial pacing in a pediatric patient following unsatisfactory outcomes with conventional epicardial leads. This adaptation represents a novel approach in the context of complex cardiac anatomy and severe post-surgical complications. The successful implementation and favorable outcome in this case may provide a new perspective for managing similar challenging scenarios where traditional pacing strategies fail.

## Conclusion

Cardiovascular surgery sometimes inevitably results in degree III AVB. Surgery is the crucial step to ensure normal epicardial electrode parameters. Proper placement of the epicardial electrode is key its safe and effective operation for better sensing, a lower threshold and longer battery life. Conventional epicardial pacemaker electrodes are usually associated with risks of high thresholds and susceptibility to fracture. Screwing in the epicardial electrode into the myocardium under the naked eye proved to be a feasible approach for implanting ventricular pacing electrodes.

## Data Availability

No datasets were generated or analysed during the current study.

## Abbreviations

AVB: Atrioventricular Block
CHD: Congenital Heart Diseases
NS: Noonan Syndrome
HOCM: Hypertrophic Obstructive Cardiomyopathy
TTE: Transthoracic Echocardiography
MRI: Magnetic Resonance Imaging
ECG: Electrocardiography
PVP: Pulmonary Valvuloplasty
